# Endostar acts as a pneumonitis protectant in patients with locally advanced non-small cell lung cancer receiving concurrent chemoradiotherapy

**DOI:** 10.1186/s12885-024-12001-6

**Published:** 2024-02-23

**Authors:** Kuifei Chen, Shuling Li, Meng Chen, Zhicheng Jin, Xuefeng Sun, Suna Zhou, Haihua Yang

**Affiliations:** 1 Taizhou hospital of Zhejiang Province, Shaoxing University, Zhejiang Province, Taizhou, 317000 China; 2 Department of Radiation Oncology, Key Laboratory of Radiation Oncology of Taizhou, Radiation Oncology Institute of Enze Medical Health Academy, Taizhou Hospital Affiliated to Wenzhou Medical University, Zhejiang Province, Taizhou, 317000 China

**Keywords:** Endostar, Radiation pneumonitis, Concurrent chemoradiotherapy, Locally advanced non-small cell lung cancer

## Abstract

**Background:**

CCRT is presently the standard treatment for LA-NSCLC. RP is one of the main obstacles to the completion of thoracic radiation therapy, resulting in limited survival benefits in NSCLC patients. This research aims to explore the role of Endostar in the occurrence of grade≥2 RP and clinical curative effect in LA-NSCLC patients.

**Methods:**

This study retrospectively analyzed 122 patients with stage III NSCLC who received CCRT from December 2008 to December 2017, or Endostar intravenous drip concurrently with chemoradiotherapy (Endostar + CCRT group). Standard toxicity of the pneumonitis endpoint was also collected by CTCAE V5.0. We further summarized other available studies on the role of Endostar in the prognosis of NSCLC patients and the incidence of RP.

**Results:**

There were 76 cases in the CCRT group and 46 cases in the CCRT+ Endostar group. In the CCRT+ Endostar group, the occurrence of grade ≥2 RP in patients with V20Gy ≥25% was significantly higher than that in patients with V20Gy < 25% (p = 0.001). In the cohorts with V20Gy < 25%, 0 cases of 29 patients treated with Endostar developed grade ≥2 RP was lower than in the CCRT group (*p* = 0.026). The re-analysis of data from other available studies indicated that Endostar plus CCRT could be more efficient and safely in the occurrence of grade≥2 RP with LA-NSCLC.

**Conclusions:**

When receiving CCRT for LA-NSCLC patients, simultaneous combination of Endostar is recommended to enhance clinical benefit and reduce pulmonary toxicity.

## Background

Lung cancer is the most common disease in terms of incidence and the leading cause of cancer death worldwide [1]. Over two-thirds of all cases of lung cancer are non-small cell lung cancer (NSCLC) [2], approximately one-third of which present locally advanced-stage disease at the time of initial diagnosis. Platinum-based chemotherapy plus concurrent radiotherapy is a standard treatment for patients with locally advanced non-small cell lung cancer (LA-NSCLC). A number of studies have shown that radiotherapy with vinorelbine combined with platinum has good efficacy. With these results, concurrent chemoradiotherapy with cisplatin and vinorelbine could be considered one of the new standard regimens for LA-NSCLC, although the employed vinorelbine doses in each phase II study were 12.5 mg/m^2^ and 15 mg/m^2^ [3–5]. Concurrent chemoradiotherapy (CCRT) followed by immunotherapy consolidation (CIT) can further improve the prognosis for LA-NSCLC patients [6]. However, radiotherapy-associated acute toxicities, especially radiation pneumonitis (RP), restrict the application of CIT after CCRT in patients with stage III unresectable NSCLC [7]. Endostatin is another broad-spectrum angiogenesis inhibitor widely used in antitumor treatment. A phase II trial HELPER study confirmed the synergistic interaction of Endostar and CCRT for patients with unresectable stage III NSCLC, and the incidences of grade 1, grade 2, and grade 3 RP were 10.4%, 7.5%, and 3.0%, respectively [8]. In addition, our previous study found that stage III lung squamous cell cancer (LSCC) patients with serum Lp (a) levels above 218 mg/L who received Endostar combined with concomitant chemoradiotherapy had longer progression-free survival (PFS) and overall survival (OS) [9]. A pooled analysis suggested that endostatin and CCRT combined to treat LA-NSCLC is effective and well-tolerated, and fewer toxicities including any grade and grade≥3 radiation pneumonitis occur [10]. Moreover, recombinant human endostatin (Rh-endostatin, or Endostar) was also identified to enhance radiosensitivity and reduce radiation-related pulmonary events in patients with advanced NSCLC [11]. Endostar was approved to apply in combination with CCRT to treat patients with unresectable stage III NSCLC in 2005 by the Chinese Food and Drug Administration.

The results from murine xenograft lung tumors showed that an anti-angiogenic drug axitinib in combination with radiotherapy could significantly decrease pneumonitis, vascular damage, and fibrosis in lung tissues [12]. Although Endostar in combination with CCRT is effective and well-tolerated, there is a lack of knowledge on whether Endostar can decrease the incidence of RP in patients more than CCRT patients treated without Endostar. Moreover, bilateral lungs receiving 20Gy(V20Gy) were an important dosimetric predictor of pneumonitis for LA-NSCLC patients treated with CCRT followed by CIT [13]. V20Gy in particular was the only factor associated with grade 2 or greater RP in lung cancer patients with NSCLC who were treated with CCRT [14]. Further, on the basis of a preliminary curative effect study [9], this study intended to explore the difference in the occurrence of grade ≥2 RP in LA-NSCLC patients treated with CCRT with or without Endostar, and the difference in LA-NSCLC patients with different V20Gy.

## Methods

### Patients

Lung cancer patients who received CCRT in Taizhou Hospital between December 2008 and December 2017 were continuously enrolled according to the following criteria: a diagnosis of NSCLC established by histology; clinical stage III according to the 8th edition of the TNM staging classification used by the International Union Against Cancer; 0-1 on the Eastern Cooperative Oncology Group's Performance Score (ECOG PS). The exclusion criteria included patients without complete radiotherapy records; patients used other anti-angiogenic drugs or lost imaging materials. The need for written informed consent was waived by the Taizhou hospital of Zhejiang Province ethics committee due to the retrospective nature of the study.

### Study design treatments and endpoints

The grouping scheme and comparative analysis were as follows.

### Chemotherapy

Vinorelbine Tartrate (Jiangsu Hansoh Pharmaceutical Group Co., LTD., Trade name: Gaenuo, Sinopharm approved H19990278) injection 12.5mg/m^2^, intravenous infusion once weekly; Carboplatin (Qilu Pharmaceutical Co., LTD., Trade name: Bobei, Sinopharm approved 20020180) injection, the area under the curve (AUC) =2, intravenous infusion once weekly; for a total of 6 cycles.

### Radiotherapy

Three-dimensional conformal radiation therapy (3D-CRT) with the radiotherapy prescription of 60 Gy/2.0 Gy/30 fractions to the planning target volume (PTV), once a day, 5 times a week.

Recombinant human endostatin (Endostar): In the CCRT + Endostar group, the administered dose of Endostar (Shandong Simcere-Medgenn Bio-pharmaceutical Co., LTD., trade name: Endostar, Sinopharm approved S20050088) was 7.5 mg/m^2^/day with 1–14 days of continuous administration. The cycle was repeated every other week for a total of 2 cycles.

### Amifostine

Grade 2 or greater RP occurred more frequently after CCRT in NSCLC patients with V20Gy greater than or equal to 25% than in patients with V20Gy less than or equal to 25% [14, 15]. In this study, patients with V20Gy ≥25% were assigned to be treated with amifostine 400 mg hypodermic injection three times per week during CCRT, while patients with V20Gy<25% were treated without amifostine (Dalian Meiluo Pharmaceutical Factory Co., LTD., Trade name: Amifostine, Sinopharm approved H20010403) during CCRT.

The patients were reexamined once a week during chemoradiotherapy, 1 month after chemoradiotherapy, and once every 3 months thereafter. The reexamination included blood routine, blood biochemistry, chest CT, and physical examination. Chest CT was interpreted by a senior radiologist, and the imaging changes were mainly limited to spot shadows, pneumobronchial signs, line shadows, lung consolidation or cellular changes in the illuminated area, and a few patients also had imaging changes outside the radiation area. Eventually, RP was confirmed by 2 radiation oncologists unaware of the patient’s information according to the Common Terminology Criteria for Adverse Events version 5.0 (CTCAE V5.0) [16]. Evaluation of grade ≥2 RP was considered symptomatic RP, which requires medical intervention or limiting instrumental activities of daily living. The primary endpoint was set as the incidence of grade 2 or greater RP.

## Statistical consideration

For description, categorical variables are reported as count and percentage. Continuous variables are reported as mean ± standard deviations. The chi-square test and Fisher’s exact test were used for categorical variables. The T test was used for continuous variables. All statistical procedures were performed with SPSS version 23.0 (SPSS Inc., Chicago, IL, USA). P < 0.05 was defined as a statistically significant difference (two-sided).

## Results

### Patients

**Fig. 1:**
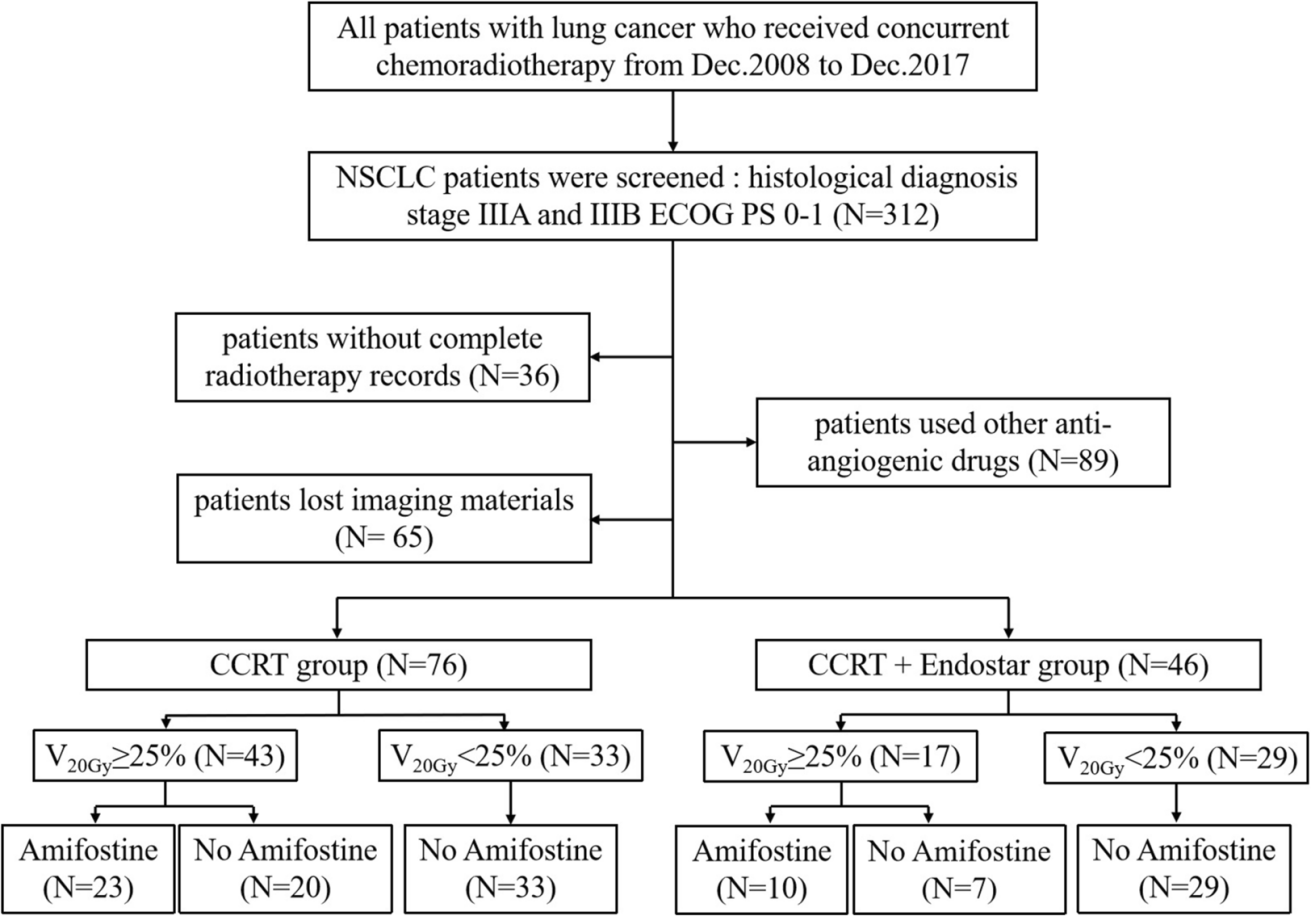
Flowchart of participants throughout the retrospective study. LA-NSCLC: locally advanced non-small cell lung cancer, NSCLC: non-small cell lung cancer, ECOG PS, Eastern Cooperative Oncology Group's Performance Score, CCRT, concurrent chemoradiotherapy

A total of 312 LA-NSCLC patients underwent CCRT from December 2008 to December 2017, of which 122 were enrolled according to the filter criteria (Fig. 1). A total of 46 patients received CCRT combined with Endostar (CCRT + Endostar group), while the others (N = 76) received CCRT alone (CCRT group). Of these, 25 patients (20.5%) developed grade ≥2 RP after treatment, 6 of which received Endostar (13.0%), and 19 of which received non-Endostar (25.0%). There was no significant difference in the patients grouped by baseline clinical factors including age, gender, smoking history, pathology, amifostine, total lung mean lung dose (MLD), clinical stage, or gross target volume (GTV) (Table 1).

**Table 1:** Baseline and clinical characteristics of patients and their correlation with Endostar

**Characteristics**	**CCRT group**	**CCRT+Endostar group**	***P*** ** Value**
Age(mean±sd)	59.08±10.062	62.15±8.602	0.357
Gender			0.911
Male	65(62.5)	39(37.5)	
Female	11(61.1)	7(38.9)	
Smoking			0.928
Non-smoker	16(61.5)	10(38.5)	
Smoker	60(62.5)	36(37.5)	
Pathology			0.844
Squamous	59(62.8)	35(37.2)	
Adenocarcinoma	17(60.7)	11(39.3)	
Stage			0.711
IIIA	34(64.2)	19(35.8)	
IIIB	42(60.9)	27(39.1)	
Amifostine			0.304
No	53(59.6)	36(40.4)	
Yes	23(69.7)	10(30.3)	
GTV(cm^3^)			0.062
<503.5(median)	43(70.5)	18(29.5)	
≥503.5(median)	33(54.1)	28(45.9)	
Total lung MLD(cGy)			0.161
<1450.7(median)	38(69.1)	17(30.9)	
≥1450.7(median)	38(56.7)	29(43.3)	
V20Gy (%)			0.036*
<25%	33(53.2)	29(46.8)	
≥25%	43(71.7)	17(28.3)	

*RP* radiation-induced pneumonitis, *CCRT* concurrent chemoradiotherapy, *GTV*, gross tumor volume, *MLD* mean lung dose

^*^ *P*<0.05

### Incidence of ≥ grade 2 RP

CCRT-cohorts were grouped by V20Gy≥25% to perform subgroup analysis (Table 2). Of the 19 patients with ≥grade 2 RP, there were 13 patients with V20Gy ≥25% and 6 patients with V20Gy <25%. The incidence of grade ≥2 RP in the patients with V20Gy ≥25% (30.2%) was greater than that in the patients with V20Gy <25% (18.2%), but without significant difference(*P*=0.229). In the CCRT + Endostar group, 6 (35.3%) of 17 patients with V20Gy ≥25% developed grade ≥2 RP, while 0 (0.0%) of 29 patients with V20Gy <25% developed grade ≥2 RP (*P*=0.001). Then, patients with V20Gy ≥25% were further grouped by treatment with or without amifostine There were 20 individuals treated without amifostine and 23 patients treated with amifostine in the CCRT group. Of 23 patients treated with amifostine, 3 (13.0%) developed grade ≥2 RP, while 10 (50.0%) of 20 patients treated without amifostine developed grade ≥2 RP (*P*=0.008). In the cohorts with V20Gy <25%, 6 (18.2%) of 33 patients treated without Endostar developed grade ≥2 RP, while 0 (0.0%) of 29 patients treated with Endostar developed grade ≥2 RP (*P*=0.026). In the cohorts with V20Gy ≥25%, 3 (13.0%) of 23 patients treated with amifostine alone developed grade ≥2 RP, while only 1 (10.0%) of 10 patients treated with amifostine plus Endostar developed grade ≥2 RP (*P*>0.05).

**Table 2:** The difference in the incidence of ≥ grade 2 RP at different subgroups

**Factors**	**Subgroup**	**No. Of grade <2RP(N)(%)**	**No. Of grade ≥ 2RP(N)(%)**	***P*** ** Value**
CCRT	V_20Gy_< 25%	27(81.8)	6(18.2)	
	V_20Gy_ ≥ 25%	30(69.8)	13(30.2)	0.229
	V_20Gy_ ≥ 25% without Amifostine V_20Gy_ ≥ 25% with Amifostine	10(50.0)	10(50.0)	
		20(87.0)	3(13.0)	0.008*
CCRT + Endostar	V_20Gy_ < 25%	29(100.0)	0(0.0)	
	V_20Gy_ ≥ 25%	11(64.7)	6(35.3)	0.001*
V20Gy < 25%	No Endostar	27(81.8)	6(18.2)	
	Endostar	29(100.0)	0(0.0)	0.026*
V20Gy ≥ 25%	Amifostine + No Endostar	20(87.0)	3(13.0)	
	Amifostine + Endostar	9(90.0)	1(10.0)	>0.05

*RP* radiation pneumonitis, *CCRT*, concurrent chemoradiotherapy

^*^ *P*<0.05

### Other studies about endostar

**Fig. 2:**
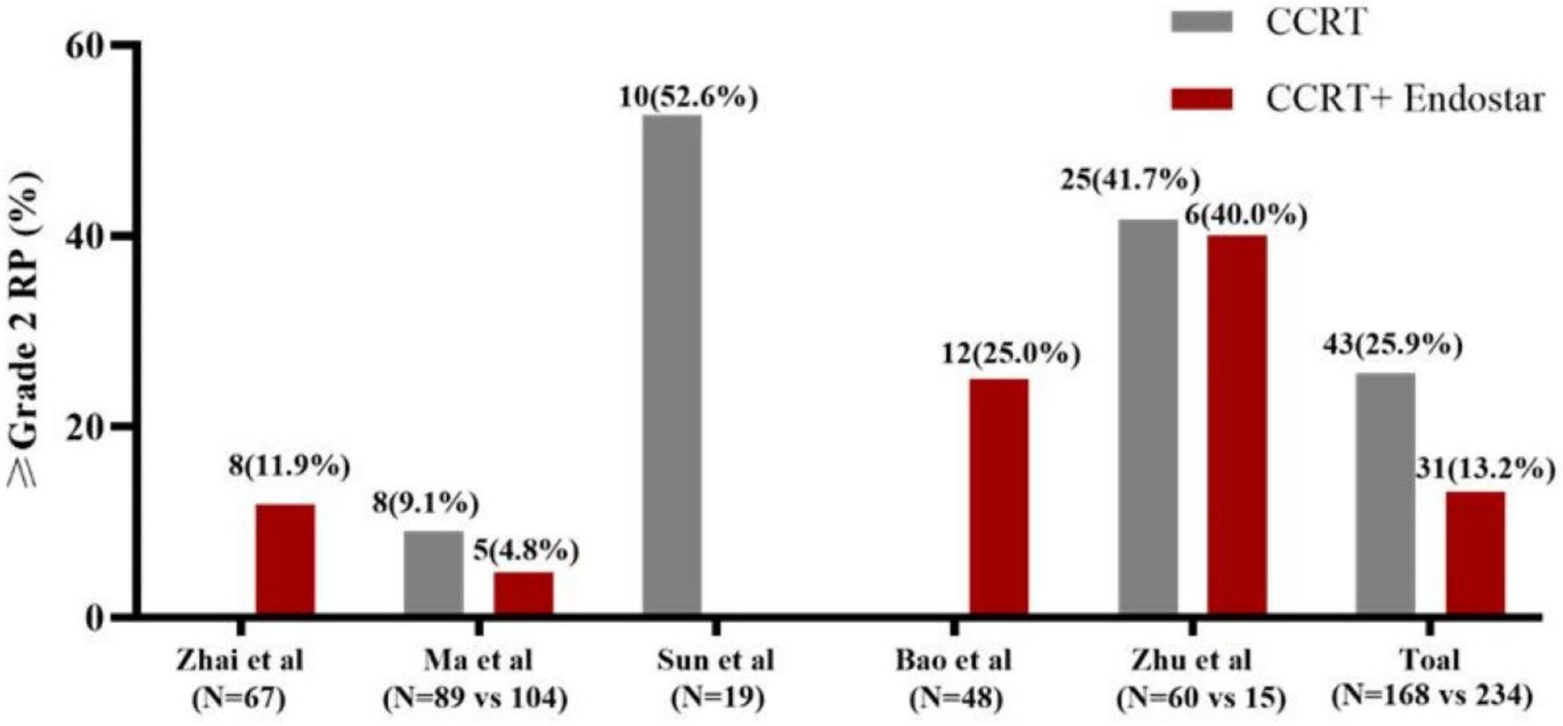
The difference in the incidence of grade ≥2 RP in the available studies. RP, radiation-induced pneumonitis; CCRT, concurrent chemoradiotherapy

The available studies on the role of Endostar in the prognosis and RP incidence of NSCLC patients are summarized in Table 3. These studies contained complete information about follow-up data and radiation pneumonitis incidence [8, 11, 17–20]. The major objects were patients with unresectable LA-NSCLC. In these studies, a total of 234 evaluable patients received Endostar combined with CCRT and 168 evaluable patients received CCRT. Patients received a total dose of 60–66Gy in 30–33 fractions for 6–7 weeks. In this study, the incidence of grade ≥2 RP was 13% in the Endostar group and 25% in the CCRT group. Compared with other studies on Endostar, the incidence of grade ≥2 RP in this study was similar. The incidence of grade ≥2 RP was on the high side at 40% in the Zhu study, considering the small sample size and some stage IV patients. Endostar addition was confirmed to improve the survival outcomes in LA-NSCLC patients receiving CCRT while presenting a downward trend in the incidence of radiation-related pulmonary events (Fig 2). And previous studies analysis indicates that Endostar combined with CCRT presents a promising treatment modality in treatment of LA-NSCLC. Combination of Endostar and concurrent chemotherapy leads to better response rate, local control rate, and survival, demonstrating superior short- and long-term survival benefits.

**Table 3:** The efficacy of endostar with NSCLC in previously reported studies

**Author**	**Patients(N)**	**Stage**	**Radiotherapy**	**Concurrent chemotherapy**	**Endostar**	**Pneumonitis(%)**	**PFS**	**OS**	**Comments**	**Reference**
Zhai et al.	67	III	60-66Gy/30–33F, 5 times /week	50 mg/m^2^/d of cisplatin on days 1, 8, 29, and 36 plus 50 mg/m^2^/d of etoposide on days 1–5 and days 29–33	7.5 mg/m^2^/day for 5 days before the beginning of RT, repeated at week 2, 4, and 6 during RT	grade≥ 2 RP 11.9%	1, 2, and 3-year PFS were 50.7%, 34.8%, and 28.2%	1, 2, and 3-year OS were 82.1%, 59.9% and 47.7%	Endostar had a better OS, promising 2year-PFS with acceptable toxicity	[8]
Ma et al.	193	III	2Gy/F/day, 5times /week	etoposide + cisplatin 50mg/m^2^ ; DP/C 65mg/m^2^ or carboplatin AUC =5; TP/C 135–175mg/m^2^,65mg/m^2^ or carboplatin AUC=5; NP/C 60mg/m^2^,65mg/m^2^; AP/C 500mg/m^2^,65mg/m^2^; GP/C 1000mg/m^2^,65mg/m^2^; repeated every 4 weeks	7.5 mg/m^2^/day with 5–7 days	(Endostar) late-stage 1 grade lung injury-14.4%, late-stage 2 grade lung injury-3.80%; (non-Endostar) late-stage 1 grade lung injury-33.7%; late-stage 2 grade lung injury-9.10%;(*P*=0.02)	NR	(Endostar) the mOS-29.7m 5year OS rate-34.7%;(non-Endostar)the mOS-21.3m 5year OS rate-23.6%;(*P*=0.038)	Endostar improved mOS and 5-year survival rate, reduced the toxic and side effects	[13]
Sun et.al	19	III	60-66Gy/30–33F,5 times /week	chemotherapy consisting of weekly 50 mg/m^2^ paclitaxel over 1 h, weekly 2 mg/mL/min carboplatin over 30 min	7.5 mg/m^2^ endostatin over 3h infusion between days 1 and 14 and between days 22 and 35	26 % had grade 3 RP	mPFS was 10.0m (95%CI:7.6-12.3m)	mOS was 14.0m (95% CI:10.7–17.2m)	endostatin's influence on NSCLC patient survival remains unknown, with unacceptable toxicity	[14]
Bao et.al	48	III	60-66Gy/30–33F for 6 -7 weeks	two doses of docetaxel(65 mg/m^2^) and cisplatin (65 mg/m^2^) on days 8 and 36	7.5mg/m^2^/d was administered over 4 h each day for 7 days at weeks 1, 3, 5, and 7	grade≥ 2 RP 25% grade> 3 RP 12%	1-, 2-, and 3year PFS rates were 48%, 27%, and 16%	1-, 2-, and 3year OS rates were 81%, 50%, and 30%	Endostar with CCRT for LA-NSCLC patients promises short-term effect and local control rates	[15]
Zhu et.al	75	III and IV	PCTV60–66Gy/30–33F/6–7 weeks for stage III, PGTV54–60Gy/27–30F/5.5–6 weeks for stage IV	NR	210mg total, continuous infusion for 5–7 days via micropump every 3 weeks	Endostar group exhibited a numerically lower rate of RP relapse, RP death and pulmonary fibrosis	mPFS was 8.0 and 4.4m (HR:0.53; 95% CI: 0.32–0.90; p = 0.019) for Endostar and non-Endostar groups	mOS was 40.0 and 13.1m (HR: 0.53; 95% CI: 0.28–0.98; p = 0.045) for Endostar and non-Endostar groups	endostatin showed better survival outcomes and a tendency toward fewer radiation-related pulmonary events in NSCLC patients	[7]

*NSCLC* non-small cell lung cancer, *RT* radiotherapy, *RP* radiation pneumonitis, *PFS* progression-free survival, *OS* overall survival, *mPFS* median progression-free survival, mOS median overall survival, *PCTV* planning clinical target volume, *PGTV* planning gross target volume, *HR* hazard ratio, 95%*CI* 95% confidence interval, *NR* not reference, *AUC* area under the curve, *LA-NSCLC* locally advanced non-small cell lung cancer

## Discussion

Radiation-induced lung injury (RILI) is one of the main obstacles to the completion of thoracic radiation therapy, resulting in limited survival benefits in NSCLC patients [21]. The acute injury stage (RP) and the chronic injury stage (radiation pulmonary fibrosis) are the major processes of RILI. Accumulated studies have identified various predictors of RP, such as smoking history, tumor location, age, and dosimetric variables of the lung (V20Gy and MLD) [22–26]. There was no significant difference in the patients grouped by baseline clinical factors including MLD. Because of V20Gy as an imbalanced risk factor and amifostine as the confounding factor in this study, stratified analysis was performed to determine the change in the incidence of grade ≥ 2 RP in different subgroups. Occurrence of grade ≥ 3 RP is the most common event to estimate radiation-induced intolerable toxicity affecting the prognosis of NSCLC patients [22, 23]. Nowadays, ICIs after CCRT are widely recommended for unresectable LA-NSCLC patients [6]. However, RP accounted for the majority of CCRT toxicities precluding the administration of ICIs and consisting mostly of grade 2 RP consisted [7, 27]. Therefore, the incidence of grade 2 or greater RP was set as the primary endpoint in this study. The dosimetric variable of the lung (V20Gy) was a significant risk factor in predicting the incidence of grade ≥ 2 RP [13, 27, 28]. Thus, we investigated the role of Endostar in LA-NSCLC patients grouped by V20Gy in this study. Our results showed that patients with V20Gy≥25% had a higher incidence of grade ≥ 2 RP.

Amifostine, a classical cytoprotective agent, significantly reduced the incidence of grade ≥2 RP in patients with V20Gy≥25%. Endostar not only reduced the incidence of grade ≥2 RP in patients with V20Gy<25% but also further enhanced amifostine-mediated reduction in the incidence of grade ≥2 RP in patients with V20Gy≥25%. Amifostine is used as a cytoprotective drug to protect normal tissue from radiation exposure, and it is preferentially accumulated in normal tissues through active transport [29]. Activated amifostine is quickly produced by vascular endothelial cell alkaline phosphatase in vivo, which converts inactive amifostine to the active free thiol and inorganic phosphate. Normal tissues preferentially take up the free thiol to eliminate irradiation-induced production of oxygen-free radicals, avoiding the damaging hydroperoxide radical-mediated DNA damage and promoting cell death [30]. Previous clinical investigations showed that amifostine may shield healthy tissues from the unexpected toxicity of radiation therapy and chemoradiotherapy without affecting anti-tumor effectiveness [31, 32]. Lung cancer patients treated with radiotherapy or chemoradiotherapy may benefit from amifostine. According to a meta-analysis [33], amifostine significantly decreased the occurrence of acute pulmonary toxicity by 44% compared to treatment without amifostine. The acute pulmonary toxicity caused by CCRT has been demonstrated to be mitigated by amifostine in many investigations [34–36]. There were similar results in the present study indicating that amifostine application significantly decreased the incidence of grade ≥2 RP across all subgroup analyses. The extra use of amifostine faces a challenge in economic costs and patient compliance. Amifostine also has side effects such as nausea, vomiting, and hypotension. It is necessary to explore the popular anti-tumor drugs that have an advantage in RP remission.

Endostar, bevacizumab, and anlotinib are the most commonly used anti-angiogenic medications for the anti-tumor treatment of lung cancer patients. Only Endostar has been approved to be applied in combination with radiotherapy or CCRT for LA-NSCLC patients based on its superior safety profile and less harmful side effects. Endostar, a C-terminal fragment naturally derived from type XVIII collagen, performs better simulations of endogenous endostatin in tumor suppression in vivo with fewer side effects than other antiangiogenic drugs. Hypoxia increased macrophage infiltration, transforming growth factor beta (TGF-β) generation, and vascular endothelial growth factor (VEGF) upregulation to form radiation damage [37]. Tanabe et al [38] found that endostatin suppressed peritoneal fibrosis by significantly reducing vascular endothelial growth factor A (VEGF-A), alpha-smooth muscle actin (α-SMA), and the pro-fibrotic factor TGF-β1 in a mouse model. Another investigation revealed that Endostar alleviated radiation-induced pulmonary alveolitis, pulmonary edema, fibrosis, and TGF-β1 release in mice compared to radiation-exposed mice treated without Endostar, suggesting that Endostar can reduce radiation-induced pulmonary lesions [39]. Clinical data also showed that Endostar safely improved the effectiveness of chemoradiotherapy (CRT) in NSCLC [8, 11, 17], and, Endostar might lessen the frequency of acute RILI by suppressing the production of inflammatory markers associated with RILI [39]. In addition, anti-angiogenic medicines can promote the normalization of tumor blood vessels within a specific time window to remodel hypoxia and the immunosuppressive tumor microenvironment [40]. Endostar can regulate metabolism and hypoxia in the tumor microenvironment contributing to synergistically im-proving the anticancer effects of radiotherapy [41]. In this study, patients in the CCRT + Endostar group of the research received treatment every other week, utilizing the effect of antiangiogenic medicines on the "normalization" of tumor blood vessels during the window period of chemoradiotherapy. We found that Endostar effectively reduced the occurrence of grade ≥2 RP in LA-NSCLC patients, which suggested that Endostar may be an effective anti-tumor drug with an advantage in RP remission and can be safely utilized in combination with other anti-tumor treatments.

In addition, Endostar combined with immune checkpoint inhibitors (ICIs) in NSCLC has attracted a great deal of attention in ongoing clinical trials. The completed studies have presented favor results (Table 4) [42–44]. The research endpoint, including the objective response rate (ORR), disease control rate (DCR), duration of response (DOR), clinical benefit response rate (CBR), PFS, OS, and adverse events (AEs), saw positive results. Above all, Endostar plus CCRT or ICIs is an effective and safe therapeutic strategy for patients with advanced NSCLC.

**Table 4:** Clinical trials about Endostar combined with ICIs in NSCLC

	**Nature**	**Inclusion criteria**	**Patients**	**Endostar**	**ICIs**	**Result**	**Reference**
Lv et.al	clinical	advanced NSCLC with EGFR(-) or ALK(-) and ineffective previous treatment	34	210 mg, continuous intravenous infusion for 168h every 4 weeks	nivolumab (3mg/kg, intravenous drip, day1) every 2 weeks	ORR 41.2%(95%CI:23.7-58.6%) DCR 64.7%(95%CI:47.8–81.6%) CBR 44.1%(95%CI:26.5–61.7%) DOR 6.9m(95%CI:4.4–9.4m) mPFS 6.8m(95%CI: 1.1–12.1) mOS 17.1m (95%CI: 6.6–27.6) grade 1-2 TRAEs 41.1% grade 3-5 TRAEs 11.8%	[19]
Wu et.al	clinical	advanced NSCLC	21	usage not mentioned	Camrelizumab	ORR 71%, DCR 100% thrombocyto-penia 24%,>3 grade 10% nausea and vomiting 24%,>3grade 5% liver damage 19% RCCEP related with camrelizumab>3 grade 0%	[17]
Wu et.al	clinical	advanced stage (IIIB and IV) NSCLC	27	210 mg, continuous intravenous infusion from day 1-3 every 3 weeks	Camrelizumab 200 mg every 3 weeks	ORR 48%, DCR 85%,CR 3.7% mPFS 8.9m anemia67%,>3 grade 11% nausea and vomiting 41%, >3 grade 4% immune-related hepatitis 3.7% RCCEP related with camrelizumab 41%	[18]

*ICIs*, immune checkpoint inhibitors, *NSCLC*, non-small cell lung cancer, *EGFR* epidermal growth factor receptor, *ALK* anaplastic lymphoma kinase, *ORR* objective response rate, *DCR*, disease control rate, *CBR* clinical benefit response rate, *DOR* duration of response, *mPFS* median pro-gression-free survival, mOS median overall survival, *TRAEs* treatment-related adverse events, *RCCEP* reactive cutaneous capillary endothelial proliferation

Moreover, anti-angiogenesis therapy performed synergistic anti-tumor effects in combination with ICIs [45–47]. Numerous clinical trials have verified that patients with advanced NSCLC can benefit from combining ICIs and anti-angiogenic medicines with an acceptable safety profile [48–51]. Lv et.al [44] proposed that Endostar combined with nivolumab as a second-line or later treatment demonstrated encouraging effectiveness and good tolerability in patients with advanced NSCLC. However, the IMPOWER 150 study [52] found that patients treated with bevacizumab combined with ICIs suffered severe side effects. These findings suggested that Endostar may be a better option in the combination treatment with ICIs and anti-angiogenesis drugs. Similarly, when anti-angiogenic drugs are combined with ICI and thoracic radiotherapy, Endostar is the best choice because it can reduce the incidence of RP and improve the anticancer effects. These findings need further validation by more multi-center double-blind randomized controlled clinical studies.

This study has some shortcomings, as follows: to begin with, it was a retrospective study with an insufficient sample size, and radiotherapy was performed by 3D-CRT treatment, not intensity-modulated radiation therapy (IMRT). IMRT is a popular radiotherapy technology applied in the clinic to enhance accuracy and reduce toxicity. We have no idea whether the advantages of Endostar will be further developed in combination with IMRT. Moreover, Endostar combined with amifostine showed a further tendency of RP reduction in LA-NSCLC patients with V20Gy≥25%. This result needs prospective randomized clinical trials for confirmation. Addtional research is needed to validate the best dosing, frequency, and duration of Endostar in combination with CCRT for the treatment of inoperable LA-NSCLC.

## Conclusions

Endostar and amifostine were shown to decrease the incidence of grade ≥ 2 RP in LA-NSCLC patients treated with CCRT. Endostar can improve the clinical benefits of CCRT while it may reduce the incidence of grade ≥2 RP. When receiving CCRT for LA-NSCLC patients, simultaneous combination of Endostar is recommended to enhance clinical benefit and reduce pulmonary toxicity.

## Data Availability

The datasets used and/or analysed during the current study are available from the corresponding author on reasonable request.

## Abbreviations

CCRT: concurrent chemoradiotherapy
LA-NSCLC: locally advanced non-small cell lung cancer
RP: radiation pneumonitis
NSCLC: non-small cell lung cancer
CTCAE V5.0: Common Terminology Criteria for Adverse Events version 5.0
CIT: immunotherapy consolidation
LSCC: lung squamous cell cancer
PFS: progression-free survival
OS: overall survival
ECOG PS: Eastern Cooperative Oncology Group's Performance Score
AUC: area under the curve
3D-CRT: three-dimensional conformal radiation therapy
PTV: planning target volume
MLD: mean lung dose
GTV: gross target volume
ICIs: immune checkpoint inhibitors
ORR: objective response rate
DCR: disease control rate
DOR: duration of response
CBR: clinical benefit response rate
AEs: Adverse Events
RILI: radiation-induced lung injury
TGF-β: transforming growth factor beta
VEGF: vascular endothelial growth factor
VEGF-A: vascular endothelial growth factor-A
α-SMA: alpha-smooth muscle actin
CRT: chemoradiotherapy
IMRT: intensity modulated radiation therapy
RT: radiation therapy
PCTV: planning clinical target volume
PGTV: planning gross target volume
HR: hazard ratio
95%CI: 95% confidence interval
NR: not reference
EGFR: epidermal growth factor receptor
ALK: anaplastic lymphoma kinase
mPFS: median progression-free survival
mOS: median overall survival
TRAEs: treatment-related adverse events
RCCEP: reactive cutaneous capillary endothelial proliferation
